# A Comparative Analysis of Maternal Nutrition Decision-Making Autonomy During Pregnancy—An Application of the Food Choice Process Model in Burkina Faso and Madagascar

**DOI:** 10.1177/03795721231217554

**Published:** 2023-12-20

**Authors:** Raphia M. Ngoutane, Laura E. Murray-Kolb, Ramakwende Zoma, Césaire T. Ouédraogo, Kesso Gabrielle van Zutphen, Rachel Bruning, Andry Razakandrainy, Elizabeth Ransom, Nita Dalmiya, Klaus Kraemer, Stephen R. Kodish

**Affiliations:** 1 The Pennsylvania State University, University Park, PA, USA; 2 Purdue University, West Lafayette, IN, USA; 3 International Rescue Committee, Bamako, Mali; 4 Sight and Life, Basel, Switzerland; 5 GRET, Antananarivo, Madagascar; 6 United National Children’s Fund, New York, NY, USA

**Keywords:** maternal nutrition, autonomy, food choice process model, qualitative research, Burkina Faso, Madagascar

## Abstract

**Background:**

Adequate nutrition has been cited as one of the most critical components for optimal health outcomes during pregnancy. Women in Burkina Faso and Madagascar experience high rates of undernutrition due to lack of knowledge, finances, cultural norms, and autonomy. Therefore, this study aimed (1) to describe typical maternal diets during pregnancy in Burkina Faso and Madagascar, (2) to understand the multilevel factors that influence women’s nutrition decision-making, and (3) to explore the extent to which women have nutrition decision-making autonomy during pregnancy.

**Methods:**

This study was conducted between October 2020 and February 2021 in Burkina Faso and Madagascar. Semi-structured interviews, focus group interviews, and free lists were conducted among women of reproductive age and pregnant and lactating women. Textual data from interviews were recorded and translated verbatim from local languages into French. The Food Choice Process Model guided textual content analysis using *Dedoose* software. Free list data were analyzed using cultural domain analysis approaches.

**Results:**

In Burkina Faso and Madagascar, women primarily consumed staple foods such as rice and tô during pregnancy. Participants cited eating fruits and vegetables when available, while the animal source foods were rarely consumed. Across both contexts, nutrition during pregnancy was influenced by factors that impact food choices, such as social factors, resources, ideals, and personal factors. While women and men in Madagascar had more shared decision-making on critical domains such as finances, men were the primary decision-makers in most areas of inquiry (eg, finances) in Burkina Faso.

**Conclusions:**

The lack of adequate diverse diet consumed during pregnancy is primarily due to important factors including social factors and resources. Understanding the ability for women to consume optimal diets during pregnancy is needed to target behavioral change in maternal nutrition programming.

## Abstract (plain language summary)

**Plain language title:**

A comparison of How Pregnant Women Make Decisions About What to Eat in Burkina Faso and Madagascar Using a Model Called the Food Choice Process Model

**Plain language summary:**

Having a proper diet is very important for the health of pregnant women. In Burkina Faso and Madagascar, many women do not get enough nutritious food due to lack of knowledge on what to eat, lack of money, cultural traditions, and not having control over their own choices. This study wants to find out (1) what women eat during pregnancy in Burkina Faso and Madagascar, (2) what influences the decisions women make about what to eat during pregnancy, and (3) explore how women had decision-making autonomy during pregnancy. The study took place in Burkina Faso and Madagascar. The researchers talked to women who could have babies and women who were already pregnant or breastfeeding. They used different methods like focus group discussions and semi-structured interviews to gather information. They recorded and translated everything that was said from the local languages to French. They used special software to analyze the information from the interviews. They also used a free list to understand the things women mentioned most often when talking about food. In Burkina Faso and Madagascar, women mostly ate basic foods like rice and tô during pregnancy. Sometimes they ate fruits and vegetables when they were available, but they did not eat much meat or other foods from animals. In both places, the women’s food choices during pregnancy were influenced by different things like what their friends and family thought, how much money they had, their personal preferences, and other factors. In Madagascar, men and women made decisions together about important things like money, but in Burkina Faso, men were usually the ones making the decisions. The lack of a proper diverse diet during pregnancy in Burkina Faso and Madagascar is primarily caused by social factors and resources. To help women make better food choices during pregnancy, it’s important to understand what affects their ability to have a healthy diet. This can help programs that aim to improve the nutrition of pregnant women by encouraging them to change their behavior.

## Background

Globally, while approximately 240 million adult women are underweight (<18.5 kg/m^2^) and 468 million suffer from anemia, a disproportionate burden exists among women of reproductive age living in low and middle-income countries (LMICs).^1-3^ In sub-Saharan Africa, specifically, anemia and underweight are persistent nutrition-related illnesses affecting both pregnant and nonpregnant women of reproductive age.^4,5^ For example, in 2021 the anemia prevalence among pregnant and nonpregnant women living in sub-Saharan Africa is 46% and 40%, respectively.^6^

While suboptimal nutrition affects general health and well-being, it has more significant consequences during pregnancy, as maternal nutritional status is important for reducing risk of adverse birth outcomes, including pre-term births and low birthweights.^7,8^ Inadequate access to nutritious foods and micronutrient supplementation during pregnancy can lead to deficiencies, including those of iron, iodine, zinc, and folic acid.^8^

Many inter-related, complex factors influence the diets and nutritional status of pregnant women in LMICs. These factors include the provision of an integrated package of nutrition counseling program, the training of health care professionals delivering counseling, and frequency of counseling sessions.^9^ While social factors, such as culturally bound food taboos (ie, food proscriptions) and food aversions influence maternal diets,^10-13^ food access challenges more greatly constrain the dietary choices of most people living in LMICs.^13,14^ Longstanding social norms may influence maternal diet quality as food-related decisions are often made at household rather than at individual level, and at the expense of those less empowered.^15^ Thus, the ability of an individual to make their own dietary decisions—the level of autonomy that they have—may be a factor that shapes individual diet quality, especially in settings where gender inequities persist.^16^

Previous studies examining women’s decision-making autonomy have found a positive relationship between higher individual autonomy and better health outcomes, overall.^17-19^ Although Demographic and Health Surveys ask questions related to decision-making in the context of health care, large household purchases, and visits to family or relatives,^17,20,21^ they do not ask specific questions focused on dietary choices or nutrition.^22^ Thus, researchers have suggested more exploratory, in-depth, and qualitative studies to investigate this domain.^17^

Therefore, we designed an exploratory study to better understand women’s nutrition decision-making, autonomy, and dietary choices during pregnancy in Burkina Faso and Madagascar, where maternal nutrition and birth outcome indicators, including high anemia rates (52.5% and 37.8%, respectively), underweight (11.8% and 13.7%, respectively), and low birthweight (13.1% and 17.1%, respectively), may be a result, at least in part, of such underlying social constructs.^23-26^ Our study had the following 3 aims for comparison between settings: (1) to describe typical maternal diets during pregnancy, (2) to understand the multilevel factors that influence women’s nutrition decision-making, and (3) to explore the extent to which women have nutrition decision-making autonomy during pregnancy.

## Method

### Study Setting

Burkina Faso is a landlocked country situated in West Africa, bordered by Mali, Niger, Ghana, Benin, Côte d’Ivoire, and Togo.^27^ It is made up of 13 regions, 45 provinces, and inclusive of 63 health districts.^28,29^ Our study was conducted in Yako and Ziniare districts. Based on the Global Gender Gap Index, an indicator that examines levels of gender equality across countries, Burkina Faso ranks 129 out of 195 countries.^30^ Nationally, nearly two-thirds of pregnant women aged 15 to 49 years have anemia.^31^

Madagascar is located in the Southeastern Africa region, east of Mozambique.^32-34^ The country is divided into 22 regions, two of which included our study sites: Vatovavy Fitovinany and Itasy. As of 2020, the Global Gender Gap Index ranked Madagascar 62 out of 195 countries.^30^ Approximately 26% of women of reproductive age are affected by undernutrition.^35^ Micronutrient deficiencies are a persistent challenge nationally: among pregnant women, 35.6% have anemia.^31^

### Research Design

Our study was part of a larger mixed-methods formative research project aimed at developing social and behavioral strategies to introduce multiple micronutrient supplements (MMS). Burkina Faso and Madagascar were chosen as 2 of 4 pilot countries, including Bangladesh and Tanzania, where MMS would be introduced through national health systems with the support of UNICEF and partners. Our analysis used data collected between October 2020 and February 2021.

### Data Collection Methods and Sampling Procedures

Prior to fieldwork, locally hired teams were trained in qualitative data collection methods and data management procedures. Firstly, the training-of-trainer sessions were conducted virtually between the remotely based research team and locally based trainers in both countries due to travel limitations during the COVID-19 pandemic. Then trainers facilitated data collection team training using a combination of didactic and experiential approaches conducted over several weeks with continual remote support provided. Lastly, the locally based teams carried out fieldwork procedures in line with study protocols.

To address the study aims, data were collected using focus group discussions (FGDs), semi-structured interviews, and free lists for triangulation purposes (Table 1).^36-38^

**Table 1.** Summary of Methods and Sample Sizes by Country.

	Semi-structured interviews (pregnant women)	Focus groups (pregnant and lactating women)	Free list (women of reproductive age)
Burkina Faso	22	6	30
Madagascar	24	6	60
Total	46	12	90

#### Focus group discussions

A FGD is a qualitative method whereby group discussion is facilitated by a moderator who asks semi-structured questions to a small group of participants who view themselves similarly to one another.^39^ We conducted focus groups in this study to understand socially normative perspectives on maternal diets in these two cultural contexts.^40^ Participants were purposefully sampled for focus groups consisting of between 6 and 13 pregnant and lactating women (PLW) based on age (between 18 to 49 years old), life stage (pregnant or lactating), and residence (living in one of the selected study districts).^41^ Questions asking about typical food consumption during pregnancy, household member roles and responsibilities, and decision-making around health, nutrition, and finances were included.

#### Semi-structured interviews

Semi-structured interviewing is a qualitative data collection method well suited for gaining deeper insights into social phenomena through individual narratives and stories.^42^ Interview participants were purposefully sampled based on the same criteria used for focus group recruitment. In each country, 24 women were sampled to reach “data saturation” of key themes.^36^

#### Free list

Free listing is a type of structured interviewing used to explore a cultural domain, in this case typical foods consumed during pregnancy and underlying cultural food rules (ie, food prescriptions and proscriptions [taboos]).^43^ In this study, we used the same criterion-based sampling strategy described above to recruit at least 30 pregnant women in each site.^43^ Responses to free list questions were recorded verbatim using local language terms and phrases on paper forms. Interviewers then asked semi-structured, follow-up probes that were recorded as field notes.

### Data Management and Analysis

#### Textual data

Textual data from focus groups and interviews were digitally recorded, translated, and fully transcribed from local languages (Malagasy in Madagascar; Mooré in Burkina Faso) into French for analysis by data collectors. Transcripts were then uploaded into *Dedoose* software for data management and textual analysis.^44^ A codebook was developed based on the interview guide contents, the guiding research aims, and the Food Choice Process Model.^45^ The Food Choice Process Model by Furst and colleagues was selected due to its ability to demonstrate various factors and processes that affect food choices.^45^ The model encompasses 3 main components: life course, influences, and personal system.^45^ These components consider an individual’s personal roles, social and cultural environments, resources, and food context, all of which shape one’s ability to make food choices.^45^ The model emphasizes the interrelationships among these factors and the pathways that contribute to an individual’s dietary choices.

The textual analysis of transcripts was conducted by one investigator, with consultation from research team members, following a multistep process. Firstly, an initial codebook was developed a priori using constructs from the Food Choice Process Model.^45^ Secondly, transcripts were read in their entirety. Thirdly, within *Dedoose*, we coded textual excerpts using the codebook but also allowing for emergent themes relevant to the guiding study aims.^46^ Fourthly, each theme and sub-theme were extracted from *Dedoose* for interpretation vis-à-vis the research aims. Finally, a pattern code map was developed to provide visual representations of the emergent themes and their interrelationships.^37^

#### Free list data

Free list data were managed and analyzed using Visual Anthropac.^47^ The saliency of listed items was calculated based on frequency of mention and average rank of items across participant responses. Field notes were thematically analyzed to help explain statistical results.

### Ethics

The study protocol was approved by The Pennsylvania State University, the Comité d’Ethique pour la Recherche en Santé (CERS) of Burkina Faso, and the Secretary General of the Ministry of Health for Madagascar. Verbal informed consent was obtained from all participants prior to data collection.

## Results

### Typical Maternal Diets During Pregnancy

In Burkina Faso, we found that the typical meal of a woman during pregnancy primarily consists of carbohydrate-rich foods such as *tô* (millet or corn flour dough mix) and vegetable sauces such as okra, baobab leaves sauce, and sorrel leaves sauces. Participants less frequently mentioned consuming fruits such as oranges and bananas, and various soups (goat and fish) (Table 2).

**Table 2.** Typical Foods Consumed by Women During Pregnancy in Burkina Faso.

Burkina Faso			
Local food term (in Mooré)	English term	Brief *emic* description of the term, from participant description perspective	Salience
*Tô* ^a^	Flour dough mix	Thick and malleable paste made of cereal (eg, sorghum, millet, or corn) by mixing flour with water and something sour like lemon or tamarind. It is mostly eaten with sauce and is available year-round.	0.789
Moui^a^	Rice	White cereal that can be eaten with many sauces or fried. It is tasty but not accessible to everyone.	0.603
Benga^a^	Beans	Food available year-round. Generally consumed with cooking oil.	0.393
Nemdo	Meat	Good [for consumption] but not accessible to everyone.	0.258
Orange	Orange	Seasonal fruit.	0.234

^a^ These top 3 foods were also salient in textual analysis of interview data.

Participants also discussed consuming beans during pregnancy; however, beans were less frequently consumed than rice and *tô*. In Madagascar, the typical meal of women during pregnancy consisted of rice and cassava. Sometimes women also mentioned consuming fruits such as bananas and mangoes (Table 3). These food choices were found to be influenced by a variety of factors that can be conceptualized using the Food Choice Process Model.^45^

**Table 3.** Typical Foods Consumed by Women During Pregnancy in Madagascar.

Madagascar			
Local food term (in Malagasy)	English term	Brief *emic* description of the term, from participant description perspective	Salience
Vary^a^	Rice	Dry rice (it is cooked until there is no water in the preparation). Women consume it to get energy and to be strong. Rice allows the fetus to be healthy.	0.778
Mangahazo^a^	Cassava	Cut [cassava] into small pieces and put sugar. She [pregnant woman] doesn’t feel very tired when she eats it. This food brings energy.	0.688
Akondro^a^	Banana	Provide vitamin and energy.	0.537
Ovy	Potatoes	It is cooked in water. This food is consumed because it provides energy.	0.475
Manga	Mango	Provides vitamins.	0.346

^a^ These top 3 foods were also salient in textual analysis of interview data.

### Multilevel Factors That Influence Maternal Diets

**Figure 1.**
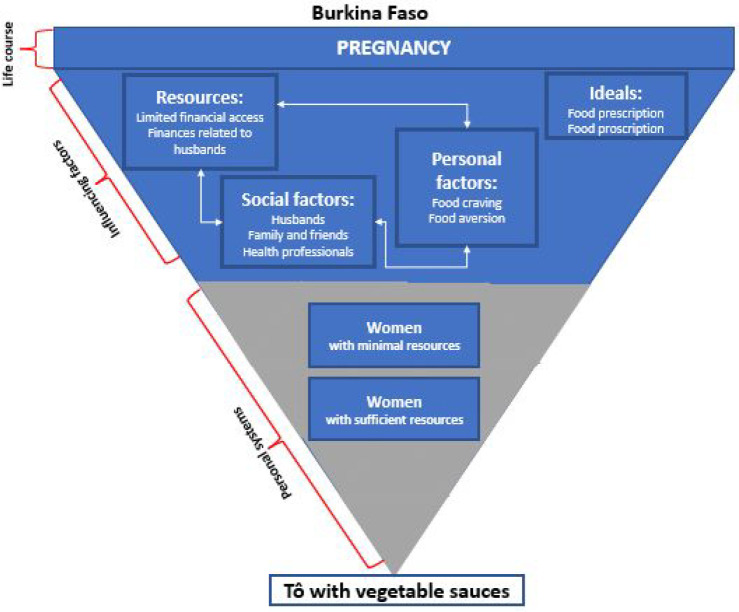
Food choice process model tailored to the Burkina Faso context.

**Figure 2.**
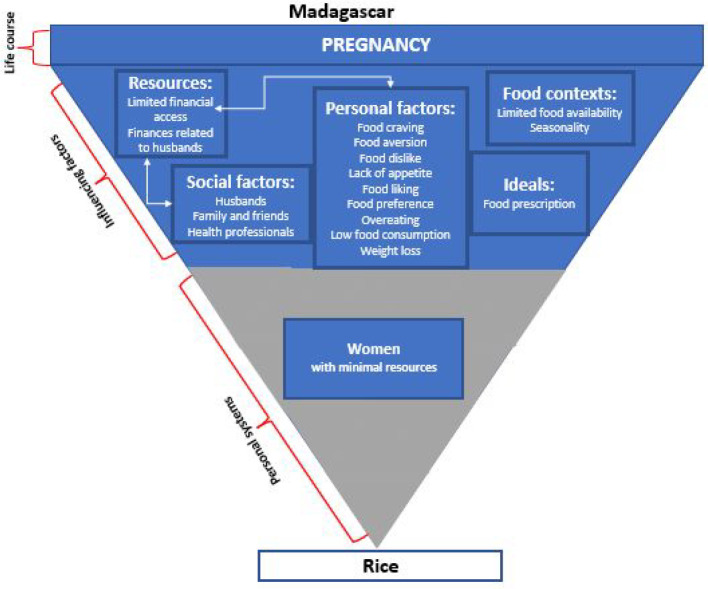
Food choice process model tailored to the Madagascar context.

Firstly, we found that social factors influence maternal nutrition choices during pregnancy in Burkina Faso and Madagascar (Figure 1, Figure 2).

Three social factors were highlighted as key players in influencing women’s nutrition decisions: (1) husbands, (2) family and friends including elder family members (eg, participant mothers and mothers-in-law), and (3) health professionals (Figure 1, Figure 2). For example, the quote below illustrates a participant’s response to her husband’s influence on what to eat,

I will cook the beans. It is my obligation to do what my husband wants. (PLW, focus group, Burkina Faso)

In addition to social factors influencing women’s nutrition decisions during pregnancy, 2 types of resources were mentioned by participants as important for dietary decisions: (1) social/interpersonal resources and (2) financial resources (Figure 1, Figure 2).

Participants frequently explained the connection between interpersonal influence of their husbands and the available financial household resources. Husbands were commonly cited as being responsible for the financial resources in the household compared to other groups. For example, when women were asked who is responsible for bringing in money to buy food at home, one pregnant woman from Madagascar mentioned that “*it is my husband who takes care of our financial needs.*” In addition to the influence of husbands on finances, another resource influencing women’s nutrition decisions was limited financial access. In Burkina Faso, women discussed the lack of food affordability and limited financial support. For example, participants explained that their consumption of *tô*, either derived from millet or corn, depended on the availability of household finances. While *tô* is generally derived from millet, participants mentioned that corn-based *tô* was consumed when there was extra money.

As you are in charge of your expenses and given enough means, if you earn 50 XOF [$0.078] then you buy either the okra or the baobab leaves for cooking. [But] you do not have enough money to buy the spices to keep them for the preparation. (PLW, focus group, Burkina Faso)

Similar to those in Burkina Faso, participants in Madagascar cited the lack of financial access as a limiting factor of adequate diets, as explained by a PLW in Madagascar who said, “*Yes, even if I want to eat chicken for example, if I can’t afford it,* [then] *I can’t eat it.*”

In circumstances where women had to choose between cooking their husbands’ preferred meal or their own, they often opted to cook both if they had sufficient resources. A woman in Burkina Faso explained, “*If you have [financial] means, you do [cook] both*.” Another participant emphasized cooking first for her husband and then cooking her meal because the husband is the head of the household.

I cook for my husband first, since he is the head of the household, before taking care of my dish. (Pregnant woman, semi-structured interview, Burkina Faso)

Dietary choices are made in participating households based not only on a woman’s ability to access specific foods but also in consideration of other household members’ preferences and requests. Women with fewer financial resources had less ability to negotiate with their husbands to make their own dietary choices. In addition to resources, a variety of personal factors were found to influence women’s dietary choices during pregnancy. Personal factors such as food cravings and aversions are connected to resources (finances) and social factors (husbands). For example, most women cited typically cooking food that their husbands preferred. However, in cases where participants craved other special foods during pregnancy, they could sometimes do so but only when they had extra financial resources. In those cases, they would prepare two separate dishes, one for themselves and one for their husband.

You will do what the husband wants…but because it is the pregnancy that [determines what you eat], if you can afford it, [then] you will do both; if not, you will bear it [his meal preference]. (PLW, focus group, Burkina Faso)

Participants also listed various personal factors during pregnancy that influence their dietary decisions. In both Burkina Faso and Madagascar, these factors included pregnancy-related food cravings and food aversions (Figure 1, Figure 2). However, the ability of women to consume those specific food cravings often depended on financial resources.

When I crave something to eat, if I have the money [then] I go shopping and come back to cook. But when I don’t have the money, [then] I cook what is available. (Pregnant woman, semi-structured interview, Madagascar)

In Madagascar only, participants also cited other personal factors influencing their diets such as particular food likes, food dislikes, and pregnancy-related lack of appetite.

In Burkina Faso, although women primarily consumed *tô*, rice and beans during pregnancy, culturally bound food prescriptions (eg, peanuts, nuts, clay, fruits, fish, and some meats) and food proscriptions (taboos) were found to influence the diets of pregnant women. Women provided cultural reasons explaining why the prescribed foods were considered healthy for pregnancy, including positive birth outcomes and maternal health. Food taboos, including eggs, pork, honey, milk, and pepper, were said to increase the likelihood of complications during birth, miscarriages, or were considered *haram* in Islam. In Madagascar, participants explained bananas, fish, and carrots to be prescribed foods with important nutrients for maternal nutrition and birth outcomes. No food proscriptions were identified in the Madagascar sample.

Lastly, in Madagascar, but not Burkina Faso, participants discussed the food environment as a factor influencing their dietary choices, specifically, seasonality and limited food availability.

### Level of Maternal Decision-Making Autonomy During Pregnancy

In Burkina Faso, we found men to be the primary household decision-makers for deciding what to eat, for permitting a woman to travel to a health center, and for spending money. For instance, women in Burkina Faso explained that their husband’s permission was important to avoid conflict and to receive money from him to pay for their own health visit.

You need to get permission from your husband to go…if you do not get permission from him, you cannot decide to go on your own because if you go without his permission, your husband might get angry. (PLW, focus group, Burkina Faso)

Women in Burkina Faso explained that decisions to take prenatal supplements (when at no cost) was their own, however. By contrast, in Madagascar, data suggest that both husbands and wives typically make such health and financial decisions jointly, although prenatal supplement consumption (when freely available) is solely up to the woman.

…when my husband comes back from work, he gives me his [earned] money…for me to go shopping at the market. (PLW, focus group, Madagascar)

Whereas male heads of households were said to largely determine dietary choices in participating Burkina Faso households, pregnant woman explained their autonomy to do so in our sample of Madagascar households.

It’s me, because my husband works all the time, and I buy everything I want to eat, he understands me because I’m pregnant, there is no problem in our home about this. (Pregnant woman, semi-structured interview, Madagascar)

Table 4 summarizes the frequency of decision-making findings by domain of interest and household member gender.

**Table 4.** Financial, Dietary, and Care-Seeking Decisions by Type of Household Member in Burkina Faso and Madagascar.

Type of decision	Burkina Faso		Madagascar	
	Decision maker	Frequency of mention	Decision maker	Frequency of mention
How to spend money	Women	3	Women	5
	Men	5	Men	5
	Others	1	Others	0
	Joint	0	Joint	4
What to eat	Women	5	Women	23
	Men	16	Men	7
	Others	1	Others	0
	Joint	0	Joint	3
Supplement consumption	Women	41	Women	31
	Men	4	Men	6
	Others	5	Others	4
	Joint	0	Joint	2
Travel to health center	Women	16	Women	15
	Men	29	Men	15
	Others	2	Others	6
	Joint	0	Joint	2

## Discussion

In both Madagascar and Burkina Faso, we found most maternal diets to be comprised of cereals such as maize, millet, rice, and cassava. However, these diets lack diversity, which is not optimal for women during pregnancy when physiological needs are higher.^48^ Specifically, a typical diet in Burkina Faso or Madagascar may provide adequate caloric density but may have limited amounts of iron, folate, and vitamin A.^49^ These findings are similar to other studies conducted in Burkina Faso and Madagascar, where authors investigated diets among women of reproductive age.^12,23,35,50-52^ Several studies also found frequent consumption of cereals such as maize, millet, rice, and vegetables for sauces.^12,50,51^ However, The World Health Organization recommends that women adequately consume various micronutrients especially vitamin A, folate, iodine, and iron during pregnancy.^8,53^ Without micronutrient adequacy during pregnancy, both maternal and child health may be compromised. Lack of adequate micronutrients can lead to several negative birth outcomes, including birth defects, preterm birth, and low birth weight.^8^ Of which outcomes such as birth defect affects 7% of newborns in Burkina Faso^54^ and 13% of newborns in Madagascar.^55^ The lack of diet diversity could be attributed to limited financial resources, Research has found that the high cost of animal-rich foods is often a barrier limiting consumption.^56^

Among many factors influencing maternal dietary choices in Burkina Faso and Madagascar, the lack of financial resources was the most salient barrier women reported. Lack of food access due to food costs relative to households’ incomes drive dietary choices across country settings globally.^35,57-59^ Individuals in both research locations noted financial limitations that impacted their capacity to acquire nutritious foods while pregnant. Consistent with our findings, previous studies in Madagascar and Burkina Faso identified a shortage of financial resources as a significant constraint, compelling participants to opt for more affordable food alternatives.^35,57-59^ The COVID-19 pandemic and associated restrictions (eg, quarantines) were on-going throughout 2020 when our fieldwork was being conducted. As a result, employment rate, availability and accessibility to foods may have been lower than usual, possibly influencing the data we collected during interviews and FGDs.^60^

Furthermore, our findings suggest that husbands have more decision-making power than their wives in participating Burkina Faso households compared to those in our Madagascar sample. Conflict avoidance was said to be a primary motivator among women in Burkina Faso when communicating with their husbands about food, health, and finances, a finding aligned with similar research across cultural settings.^61-63^ Furthermore, in Burkina Faso, a country with approximately 63% Muslims,^27^ it is possible that traditional Islamic principles may shape household decision-making patterns as well. Generally, Muslim women may be more likely to have lower decision-making power than women of other faiths, particularly in Burkina Faso where religious observance is greatly conservative.^21^ In Madagascar, where households were headed by women and Christianity was primarily practiced, it is therefore less surprising that participating women had greater decision-making autonomy around diets and health than those of Burkina Faso.^21,64^

This study had some limitations. Firstly, data collection occurred during 2020 when COVID-19 prevented travel by the study team to either country. However, partnering with local collaborators in each setting allowed for the study to be conducted as planned. Secondly, the stepwise process of translation and transcription from Mooré and Malagasy languages to French for analysis likely contributed to losses of meaning and richness during the process. Lastly, typical food consumption during pregnancy was ascertained using free lists and semi-structured interview data. Using a 24-hour dietary recall would have been a more valid and comprehensive method for assessing maternal diets.^65^

Despite the limitations, this study had several strengths. Firstly, the Food Choice Process Model served as the guiding theoretical framework of this study, allowing us to develop evidence-informed data collection instruments and analytic procedures to understand dietary choices.^36,38^ Secondly, multiple data collection methods were used during fieldwork in a form of methodological triangulation, thus generating different forms of data and perspectives to comprehensively answer the guiding research questions.^37,38^ Lastly, multiple individuals, including locally based investigators from both Burkina Faso and Madagascar, collaborated to interpret study findings in a form of analytic triangulation.^66^

## Conclusion

This study revealed that the main determinants influencing maternal dietary choices in Burkina Faso and Madagascar are financial resources and social factors. Furthermore, men were found to be the primary decision makers in Burkina Faso compared to our Madagascar sample. Understanding how and why household decisions around finances, health, and nutrition are made or not made by women in these two communities may help to inform tailored programs aiming to facilitate positive behavior change in those domains. As such, future studies must highlight such complexity by using a triangulation of data collection methods that typical approaches, such as a survey, may not be able to capture. Maternal autonomy surrounding nutrition decisions does not exist in a vacuum but is influenced differentially across settings by longstanding social and cultural norms including food taboos and gender norms. The next phase for autonomy in nutrition programming involves designing interventions that empower women to make informed dietary choices during pregnancy. This includes incorporating educational strategies to enhance nutritional literacy to reduce food taboos and increase dietary diversity, and advocating for the sensitization of men’s nutrition counseling that emphasizes the significance of optimal maternal nutrition.

## Supplemental Material

Supplemental Material, sj-pdf-1-fnb-10.1177_03795721231217554 - A Comparative Analysis of Maternal Nutrition Decision-Making Autonomy During Pregnancy—An Application of the Food Choice Process Model in Burkina Faso and Madagascar Supplemental Material, sj-pdf-1-fnb-10.1177_03795721231217554 for A Comparative Analysis of Maternal Nutrition Decision-Making Autonomy During Pregnancy—An Application of the Food Choice Process Model in Burkina Faso and Madagascar by Raphia M. Ngoutane, Laura E. Murray-Kolb, Ramakwende Zoma, Césaire T. Ouédraogo, Kesso Gabrielle van Zutphen, Rachel Bruning, Andry Razakandrainy, Elizabeth Ransom, Nita Dalmiya, Klaus Kraemer and Stephen R. Kodish in Food and Nutrition Bulletin
