# Supplementary material for: A Comparative Analysis of Maternal Nutrition Decision-Making Autonomy During Pregnancy—An Application of the Food Choice Process Model in Burkina Faso and Madagascar
Source: Food Nutr Bull. 2023 Dec 20;45(1):47–56. doi: 10.1177/03795721231217554 (PMC11047013; doi:10.1177/03795721231217554)
Supplement: Supplemental Material, sj-pdf-1-fnb-10.1177_03795721231217554 - A Comparative Analysis of Maternal Nutrition Decision-Making Autonomy During Pregnancy—An Application of the Food Choice Process Model in Burkina Faso and Madagascar [file sj-pdf-1-fnb-10.1177_03795721231217554.pdf]

	No.	Brief Code Name	Full description of code	When to use the code
**1.0 Diets prior and during pregnancy**				
1	1.1	**Diets of non-pregnant women**	Description of daily dietary consumption of non-pregnant women	Use this code when participants are discussing what non-pregnant women consume
2	1.2	**Diets of pregnant women**	Description of daily dietary consumption of pregnant women	Use this code when participants are discussing what pregnant women consume
3	1.2.1	**Healthy foods**	Description of healthy foods pregnant women consume	Use this code when participants are discussing healthy foods consumed by pregnant women (i.e.: when participants link foods to vitamins)
**2.0 Influencing factors on decision-making with regard to supplement and food choice**				
4	2.1	**Ideals**	Description of when ideals or beliefs influence decision-making	Use this code when participants discuss expectations, standards, hopes and beliefs (cultural and symbolic factors)
5	2.2	**Personal factors**	Description of Personal factors to food choice	Use this when participants discuss items including likes/dislikes, individual food styles, demographic factors (age, health status, gender, sensory preferences), state of hunger, cravings, food preferences, aversions influence decision-making
6	2.3	**Resources (finances)**	Description of when resources influence decision-making	Use this code when participants are discussing when finances, skills, and time influenced decision-making
7	2.4	**Social Framework**	Description of when family and other individuals influence decision-making	Use this code when participants are discussing when family and other individuals influence decision-making
8	2.4.1	**Men influence the decision**	Description of when men influence decisions	Use this code in situations when men (husbands or partner) influence decisions
9	2.4.2	**Others influence the decision**	Description of when other individuals influence decisions	Use this code in situations of when other individuals (e.g: in-laws, other family members, friends, doctor, religious figures) influence decisions
10	2.5	**Food context**	Description of when food related factors influence decision	Use this code participants discuss physical surrounding and social climate, availability, seasonal or market factor
**3.0 Decision-making with regard to supplement and food choice**				
9	3.1	**Women make decisions**	Description of situations when women decide on diet	Use this code in situations when women make decisions

			and supplement related decision	
10	3.2	**Men make the decision**	Description of situations when men decide	Use this code in situations when men (husbands or partner) make decisions
11	3.3	**Couples jointly make the decision**	Description of situations when decisions are made jointly by couples	Use this code in situations when couples jointly make decisions
12	3.4	**Others influence the decision**	Description of when other individuals influence decisions	Use this code in situations of when other individuals (e.g: in-laws, other family members, friends, doctor, religious figures) influence decisions
